# The porosity of felsic pyroclasts: laboratory validation of field-based approaches

**DOI:** 10.1007/s00445-023-01679-4

**Published:** 2023-10-31

**Authors:** Alessandro Pisello, Ulrich Kueppers, Kai Düffels, Paraskevi Nomikou, Donald B. Dingwell, Diego Perugini

**Affiliations:** 1 Department of Earth and Environmental Sciences, Ludwig-Maximilians-Universität München, Theresienstrasse 41/III, 80333 München, Germany; 2 Department of Physics and Geology, Universitá di Perugia, via Alessandro Pascoli, 06123 Perugia, Italy; 3 Microtrac Retsch GmbH, Retschallee 1-5, 42781 Haan, Germany; 4 Department of Geology and Geoenvironment, National and Kapodistrian University of Athens, Athens, Greece

**Keywords:** Explosive eruptions, Pyroclast analysis, Porosity, Physical volcanology

## Abstract

Volcanic eruptions are driven by magma rising through Earth’s crust. The style of an eruption depends on intrinsic and extrinsic parameters and is commonly a dynamic process. Thorough and holistic investigation of the related products is key to understanding eruptive phenomena and assessment of volcano-specific hazards. Models of such phenomena are constrained by quantification of the dispersal, the grain size distribution, and pyroclast textures. Pyroclast texture may be described in part by measurements of density and porosity, which depend on pyroclast volume determination. Yet volume determination of irregularly shaped pyroclasts cannot be achieved with geometrical laws, instead necessitating the use of alternative methodologies. Here, we test three methodologies to quantify pyroclast volume on a set of clasts collected from the Minoan eruption deposits from Santorini, Greece. We compare (1) a manual method for obtaining the lengths of three orthogonal axes of the pyroclast with a caliper, (2) an optical method to measure the longest and shortest axes of the pyroclast via multiple photographs, and (3) an Archimedean buoyancy-based method. While the optical and manual methods provide almost identical values of pyroclast volume when tested under laboratory conditions, there is a discrepancy between these two methods and the Archimedean method, which produces an overestimation of ca. 13% in volume. This discrepancy has little impact on the subsequent assessment of porosity and density for which the natural variability of values is observed to be broader. We therefore propose using the manual approach in the field as a simple and fast, yet reliable, method to obtain large volumes of quantitative data on the texture of erupted products, and we also provide a correction factor for in-field volume assessment of rhyodacitic pumices.

**Supplementary Information:**

The online version contains supplementary material available at 10.1007/s00445-023-01679-4.

## Introduction

A pivotal component of volcanic hazard assessment is to understand what controls the eruptive style of an eruption and its variability through time (Cassidy et al. 2018). Volatile budgets and outgassing efficiencies clearly play a large role in eruption style (Houghton and Wilson 1989; Mueller et al. 2011). The development of magma permeability, strongly linked to the evolution of porosity, is a further crucial factor that controls fragmentation and therefore eruptive style and the texture of erupted products (Cashman 2004; Mueller et al. 2008; Wadsworth et al. 2016; Kushnir et al. 2016; Colombier et al. 2017a). Further potential complexities that control pyroclast textures include magma deformation, post-fragmentation pyroclast welding or ongoing vesiculation, and/or contact with external water (Houghton and Carey 2015; Mitchell et al. 2019; Giachetti et al. 2021). During magma ascent, volatile exsolution following supersaturation leads to substantially overpressured bubbles (the driving force by which individual bubbles grow) where bubble growth and/or outgassing by permeable flow are limited by the increasing viscosity of the melt (Shea et al. 2010; Moitra et al. 2013). Fragmentation efficiency is inversely proportional to the size of clasts generated by an eruption, and it depends on the available energy for fragmentation, stored as gas under overpressure in a given volume of pore space (Kueppers et al. 2006a).

The porosity and permeability as well as textural properties such as bubble shape, interconnectivity, and tortuosity of pyroclasts produced by an eruption are in part a record of the amount of gas present in the system during an eruption and its dynamics. At sufficiently high cooling rates and viscosities, erupted pyroclast textures approximate the state of magma at brittle fragmentation, where shear deformation and gas overpressure could no longer accommodated by viscous flow and outgassing (Kueppers et al. 2006b). The limiting size of clasts that reliably preserve pre-fragmentation textures likely depends on the magma composition, largely through its influence on viscosity and thermal conductivity (Moitra et al. 2013; Gurioli et al. 2018). Porosity and permeability of magma change during its ascent history through variable stages of volatile oversaturation, bubble growth and coalescence, and gas/bubble loss (Houghton and Wilson 1989; Rust et al. 2004; Rust and Cashman 2011; Gonnermann and Manga 2013). Several intrinsic (e.g., chemical composition) and extrinsic (e.g., magma ascent rate) processes influence the textural and chemical evolution of magmas (Piochi et al. 2005; Noguchi et al. 2006; Ross et al. 2021; Bernard et al. 2022). Vertical and horizontal gradients of magma textures may also strongly control their response during eruption (Kueppers et al. 2015; Trafton and Giachetti 2021).

Thus, explosive eruptions produce deposits whose textural, physical, and compositional characteristics exhibit kinematic records of the sum of their eruptive and transportation paths before their final emplacement. Constraining the magnitude of past explosive eruptions (even those observed) requires the quantification of erupted tephra volume (including deposits of pyroclastic density currents) as well as its spatial distribution. In addition to stratigraphic approaches (layer/bed thickness and grain size analysis), the parameterization of eruption dynamics requires an efficient method for determining the density/porosity of juvenile volcanic particles, for which it is necessary to perform grain size analysis. As the shape of volcanic clasts is commonly irregular, any size/shape analysis technology has its limitations (see Buckland et al. 2021, for an overview).

Medium-sized lapilli clasts (16–32 mm) are commonly used in classical studies that target a textural description of eruption products (Houghton and Wilson 1989; Taddeucci and Wohletz 2001; Mitchell et al. 2019; Trafton and Giachetti 2021). Post-fragmentation vesicle growth is assumed to be of minor (or at least tolerable) importance (Todde 2023).

Quantifying the porosity of rocks requires measurement of clast mass and volume, and groundmass density. While grain size analysis is straightforward, determination of pyroclast porosity is more complex as it requires volume determination of geometrically irregular bodies. Several methods are used in the literature: (1) water displacement following Archimedes’ Principle, involving measuring the clast weight under water and in air after wrapping particles in foil or wax (Houghton and Wilson 1989; Shea et al. 2010), or evacuation in plastic bags (Kueppers et al. 2005; Shea et al. 2010). For pumice samples (with a density of <1 g/cm^3^), volume determination via water displacement requires additional sinkers and corrections for weight and volume. (2) monodisperse sphere displacement (e.g., using Geopyc 1360 Envelope Density Analyzers of Micomeritics^®^), which involves measuring a reference container full of beads with and without a pyroclast (Kawabata et al. 2015; Thivet et al. 2020b; a). This technique is adapted for microporous pumice where the number of spheres entering bubbles at the sample surface is insignificant. (3) Optical methodologies via dynamic image analyses, hereafter termed DIA (Trafton and Giachetti 2021), which involves repeated measurements of the longest and shortest axes of an individual clast to calculate the volume assuming the shape of a spheroid.

Most of these measurements have been or can exclusively be performed in the laboratory, yet we aim to develop a technique that allows for rapid and reliable measurements directly in the field to avoid the logistics involved in the measurement of large sample sizes and post-sampling shape changes due to breakage or abrasion.

## Methodology

In this laboratory study, we constrained the volume of lapilli-sized pyroclasts using the three different methodologies described above to determine a reliable, yet fast technique to constrain directly their porosity in the field. The case study here involved pumice lapilli from the Minoan eruption (VEI 7, 1650 BC) on Santorini Island, Greece (Hammer et al. 1987; Druitt et al. 1999; Johnston et al. 2014; Heiken Jr. and McCoy 2014). Clasts from volcanic deposits may exhibit different degrees of absorbed humidity and, to this end, we have investigated the influence of variable amounts of absorbed water on the porosity evaluation.

Here, we present the results of three independent methods for volume determination. The first methodology (hereon: manual method) manually measures three orthogonal axes of any clast (longest, shortest, intermediate: *a* ≠ *b* ≠ *c*) with a caliper and calculates the volume under the approximation of a three-axial ellipsoid shape. The second methodology (hereon: optical method) evaluates the longest and shortest axis of individual clasts in shadowgraphs using dynamic image analysis (DIA) instruments. Adapted software and algorithms are used to calculate the volume assuming a spheroid shape (*a* ≠ *b* = *c*). The third methodology is the Archimedean method, comparing the weight of individual clasts in air and under water; in the second case, clasts were wrapped in parafilm to prevent water from entering pores.

### Sample collection

**Fig. 1:**
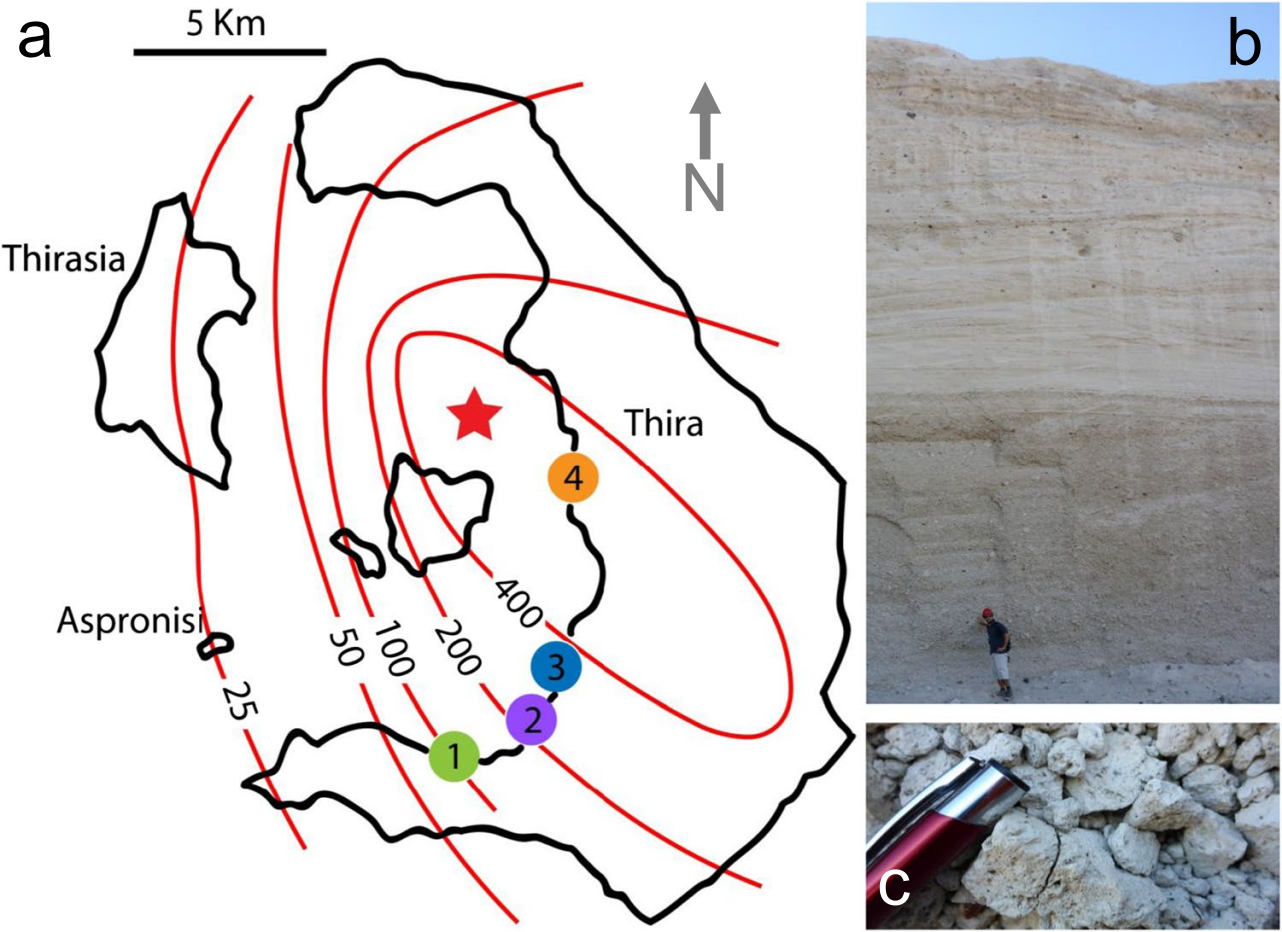
**a** Map of Santorini Island with sampling locations. **b** Example of MA (fall deposit, below) and MB ( PDC deposits, above) deposits at location 2. **c** Example of clast-supported MA deposit at location 3

The case study involves rhyodacitic pumice lapilli from the Minoan Eruption on the island of Santorini, Greece. For this eruption, stratigraphy is traditionally divided into 4 subunits (Druitt et al. 1999). The first two subunits, Minoan A (MA) and Minoan B (MB), host the pumiceous lapilli this study focuses on. MA consists of a proximal fall deposit of poorly sorted angular pumice, ranging in size from few millimeters to 15–20 cm (Fig. 1c). MB consists of cross-stratified ash-lapilli tuffs containing abundant ballistic blocks. Pumice lapilli are present and concentrated in lenses with a clast-supported fabric (Bond and Sparks 1976; Druitt et al. 1999). These deposits were selected as a target of this study because of the excellent exposure and wealth of published data on physical characteristics (Taddeucci and Wohletz 2001, defined a small density variation of +/− 0.25 g/cm^3^).

Approximately 800 clasts were hand-picked at four locations (Fig. 1a) on the island of Santorini (site 1: Akrotiri peninsula, site 2: old Megalochori quarry, site 3: Megalochori coast, site 4: Fira Quarry) where both MA and MB are present. Clasts were selected randomly by two of the authors to avoid a possible personal bias (prefix “AP” and “UK” in sample name present in Supplementary Material), wrapped individually in soft paper, and put into rigid plastic containers for transport to enable preservation of pristine clast shapes. In the lab, each clast was washed and brushed carefully to remove adhering ash, dried at 90°C for 12 h, and weighed on a precision scale (with a resolution of 10^−4^g). During this procedure, 8 clasts broke and were therefore not useful for physical property characterization.

### Volume determination methodologies

The sample set comprises clasts with the longest axis ranging between 10 and 50 mm. Here, we present three approaches for volume determination of clasts with irregular shapes, two of them relying on length measurement for volume determination and one relying on Archimedes’ principle. We divide the methods into two groups: methods that can be performed in the field and methods that can only be performed in the laboratory. In total, 794 clasts were collected and investigated by the manual method. This first investigation subsequently allowed us to select a smaller subset of clasts (*n* = 120) whose density and porosity values were representative of the whole deposit. For the Archimedean method, a further subset of clasts (*n* = 94) was selected.

#### Manual method

The manual method for the determination of *V*_m_ can be performed anywhere and is entirely suitable for fast field analyses: three orthogonal axes of each clast (*a*, *b*, *c*) are measured using an electronic caliper and the volume of an ellipsoid enclosing the clast is calculated through Eq. 1:

1 $${V}_{\textrm{m}}=\frac{\pi }{6} abc$$

In this equation, *a*, *b*, and *c* represent the longest, intermediate, and shortest axes of the ellipsoid, respectively. As the definition of the axes *a*, *b*, and *c* is potentially biased by the operator, the following protocol was introduced: the longest axis *a* is the greatest measurable length of a clast. The intermediate axis *b* is the longest measurable axis orthogonal to *a.* The shortest axis *c* is the shortest measurable length orthogonal to the *a*–*b* plane. The measurement time required per clast is approximately 15 s.

#### Optical method (dynamic image analysis)

A subset of 120 clasts was characterized using dynamic image analysis (DIA) according to ISO 13322-2. For this purpose, a CAMSIZER P4 particle analyzer (Microtrac Retsch® GmbH) was used to perform optical measurements of the clasts. The device captures 2D pictures of a falling clast and automatically retrieves the length (longest axis) and width (longest measurable axis orthogonal to length) of each clast. We discovered empirically that performing 20 repetitive drop experiments reliably determined length and width, and this number of repeats is sufficient for volume determination of clasts using DIA (Trafton and Giachetti 2021). To avoid clast break up due to impact energy when landing, an energy-absorbing landing pad was added. Clast volume was calculated assuming a spheroid shape (*V*_o_) according to Eq. 2:

2 $${V}_{\textrm{o}}=\frac{2}{3}\pi l{\left(\frac{w}{2}\right)}^2$$

where *l* is the largest measured length value and *w* is the mean width value among the 20 measurements for each clast. The measurement time required per clast is approximately 60 s.

The 20 silhouette photographs additionally allowed for calculating, with the software ImageJ, the morphometric parameters roundness (*R*), form factor (*F*), and solidity (*S*) as follows:

3 $$R=4\frac{\textrm{Area}}{\pi {l}^2}$$

4 $$F=4\pi \frac{\textrm{Area}}{{\textrm{Perimeter}}^2}$$

5 $$S=\frac{\textrm{Area}}{\textrm{Convex}\ \textrm{Area}}$$

Form factor is also referred to as circularity, but we have chosen this term for consistency with other methodological studies on volcanic clasts (Guimarães et al. 2019; Hornby et al. 2020).

#### Archimedean method

A set of 94 clasts, each individually wrapped with Parafilm^®^ to avoid the uptake of water, were weighed in air and in water, the latter through forcing its immersion with a sinking system. As the density of water at room temperature is 1 g/cm^3^, Archimedes’ principle constrains the volume of clasts (*V*_a_) through the weight reduction under water (1 g weight reduction per 1 cm^3^ water displaced). The difference of the weighed masses was then used to retrieve the volume of each clast following the equation:

6 $${V}_{\textrm{a}}=\Delta {V}_l=\frac{\Delta {M}_S}{\rho_l}$$

where ∆*M*_*S*_ is the difference between mass values of wrapped clasts measured in air and in water, *ρ*_*l*_ is the density of water, and ∆*V*_*l*_ is the volume of displaced water, which is equal to the volume of the wrapped clast. A measurement time of approximately 2 min per clast is required.

### Pycnometric studies and the assessment of density and porosity

A helium pycnometer Quantachrome^©^ Ultrapyc 1200e at LMU Munich was employed to measure the density of individual clasts and the density of rock powder, the latter to be used as dense rock equivalent (hereon DRE). Samples of precisely known weight were inserted in a measuring cell of known volume and pressurized by Helium to a predefined pressure. The precise amount of gas required to achieve this final pressure is used to calculate pycnometric volume *V*_pyc_, which represents the volume of each clast excluding the volume of interconnected pores. For each sample, five consecutive pycnometrical measurements with deviation < 0.5 % are used to average the final result. The measurement time required for each sample is approximately 10 min.

For DRE density (*ρ*_DRE_), we randomly selected clasts from MA (*n* = 15) and MB (*n* = 30) formations that were ground to grain size < 10 μm. Determination of *ρ*_DRE_ was then performed with the pycnometry protocol described above.

These measurements are then used to determine the different physical properties of the investigated clasts. The connected porosity will be calculated as follows:

7 $${\phi}^{\prime }=1-\frac{V_{\textrm{pyc}}}{V}$$

where the volume in the denominator position can be *V*_m_, *V*_o_, or *V*_a_. We distinguish connected porosity calculated using different volume values by referring to them as *ϕ*′_m_, *ϕ*′_o_, or *ϕ*′_a_.

In the same way, bulk density values *ρ*_m_, *ρ*_o_, and *ρ*_a_ will be defined using three different volume values following the equation:

8 $$\rho =\frac{m}{V}$$

where *m* is the mass of each clast.

Finally, from the *ρ*_DRE_ determination, it was possible to retrieve, assuming a constant DRE for all the clasts, different bulk porosity values for each volume determination technique, again expressed as *ϕ*_m_, *ϕ*_o_, and *ϕ*_a_ and calculated as follows:

9 $$\phi =1-\frac{m}{\rho_{\textrm{DRE}}V}$$

In this way, pycnometry analyses will show how different volume determination techniques influence the assessment of bulk density, connected porosity, and bulk porosity.

### Influence of sample humidity on porosity determination

Porous clasts from natural deposits tend to adsorb water (Giachetti et al. 2015). To assess the reliability of evaluating the porosity of pyroclasts directly in the field using the manual methodology for volume (*V*_m_) determination, we compared the dry and humid weights of pumice clasts (*n* = 50) and calculated the density and porosity of each clast using Eqs. 8 and 9. Each clast was independently dropped into water (from 15-cm height) and taken out after floating for 5 s. Dry and humid weights were measured on a precision balance (10^−4^ g).

## Results

All measured and calculated parameters are reported in the Supplementary Material. As mentioned in the previous section, a first characterization of the complete set of 794 clasts was performed using the manual methodology, from which it was possible to select a smaller representative subset of clasts to be characterized with the three methods, which initially contained 120 clasts, but this decreased to 94 because of clast breakage during optical measurements. In Table 1, we report the average, median, and standard deviation values for *V*_m_, *ρ*_m_, and *ϕ*_*m*_ retrieved from the total set of 794 clasts and on the subset of 94 clasts.

**Table 1:** Comparison of the physical properties obtained with manual method on the entire set of 794 clasts and on the smaller subset of 94 clasts

	*V*_m_ (cm^3^)		*ρ*_m_ (g/cm^3^)		*ϕ*_m_ (%)	
	794 clasts	94 clasts	794 clasts	94 clasts	794 clasts	94 clasts
Average	4.67	4.30	0.43	0.44	82.22	81.94
Median	3.16	3.64	0.42	0.42	82.56	82.53
St.Dev.	4.22	2.95	0.11	0.12	4.44	4.90

From Table 1, it can be noted that clasts from the subsets were slightly smaller than those for the manual method due to size limitations in the Camsizer. However, care was taken to select clast representative of textural variability. Overall, we obtained discrepancies on the order of 0.1 g/cm^3^ for density values, and smaller than 0.4% for porosity values.

For the sake of completeness, we report all the acquired data in the Supplementary Material, but in the following subsections, figures, and tables, we will only compare data which were obtained from the smaller subset, which was characterized with all three methodologies, i.e., 94 clasts.

### Volume

**Fig. 2:**
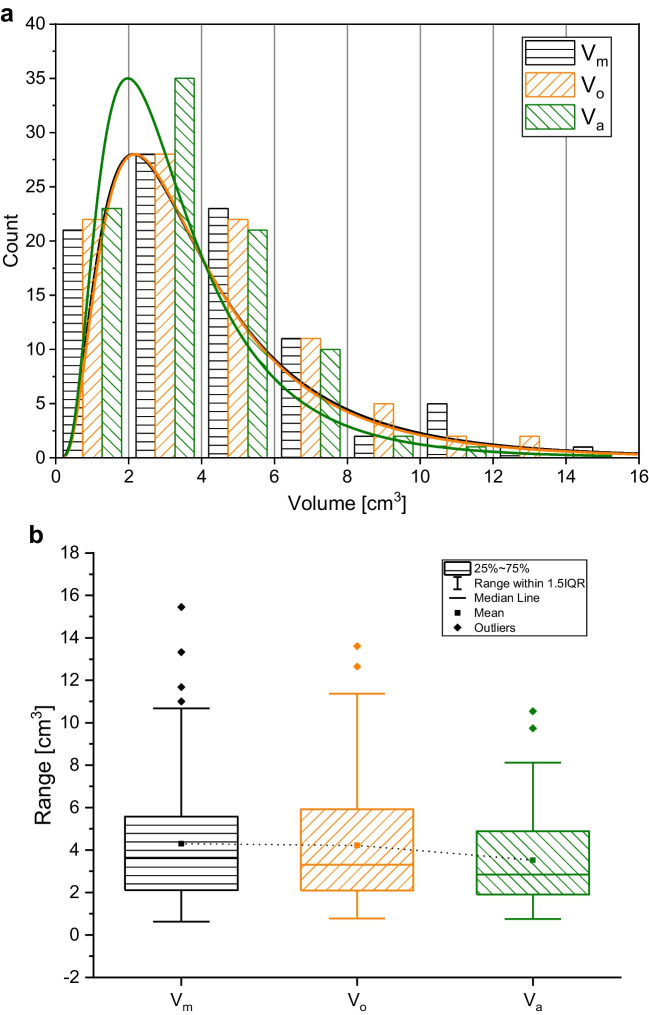
**a** Frequency distribution (bin size 2 cm^3^) of volume values (94 clasts) as retrieved with three different methodologies: manual, optical, and Archimedean. **b** Boxplot representation of the variability of the results. Manual and optical results are quasi-identical, while the Archimedes results show well-defined yet small difference

Applying three different methodologies of volume quantification to the same sample set allows constraint on the potential differences between *V*_m_, *V*_o_, and *V*_a_ (Fig. 2a). In general, manual (*V*_m_) and optical (*V*_o_) analyses result in higher values (13% on average) of volume for most clasts when compared to the Archimedean method (*V*_a_). This discrepancy is also observable for the average and median values of volume in Table 2, for which discrepancies of up to 0.8 cm^3^ (18%) were observed between *V*_m_ and *V*_a_.

**Table 2:** Comparison of the physical properties obtained with the three different methods. Statistics were calculated on the subset of 94 clasts

	Average	Median	St.Dev.
*V*_*m*_ (cm^3^)	4.30	3.64	2.95
*V*_o_ (cm^3^)	4.22	3.31	2.77
*V*_a_ (cm^3^)	3.52	2.85	2.11
*ρ*_m_ (g/cm^3^)	0.44	0.42	0.12
*ρ*_o_ (g/cm^3^)	0.44	0.42	0.12
*ρ*_a_ (g/cm^3^)	0.51	0.49	0.13
*ϕ*_m_ (%)	81.94	82.53	4.90
*ϕ*_o_ (%)	81.87	82.68	4.91
*ϕ*_a_ (%)	79.01	79.71	5.22
*ϕ*′_m_ (%)	75.72	75.98	6.52
*ϕ*′_o_ (%)	75.67	75.41	6.32
*ϕ*′_a_ (%)	71.80	71.41	7.04

We model the observed distributions using lognormal curves (Pioli et al. 2019) and observe consistent peak values at ~2 cm^3^ (Fig. 2a). The *V*_m_ and *V*_o_ curves overlap, while the *V*_a_ curve exhibits fewer coarse clasts and confirms the generally smaller volumes achieved for clasts with this methodology. This finding is confirmed when displaying the data as boxplots that show very similar distributions of *V*_m_ and *V*_o_ and minor deviation for *V*_a_ (Fig. 2b).

On average, the parafilm wrapping represented 12.8 wt.% of the bulk (clast + parafilm) weight. Assuming—for simplicity—that parafilm maintained its density after stretching, this would translate to a maximum possible volume overestimate of 6.6%. Accordingly, the Archimedean method should slightly overestimate the real volume of a clast. Figure 2, however, reveals this overestimation is considerably smaller than the overestimation caused by shape approximation which led to the determination of *V*_m_ and *V*_o_.

### Shape

**Fig. 3:**
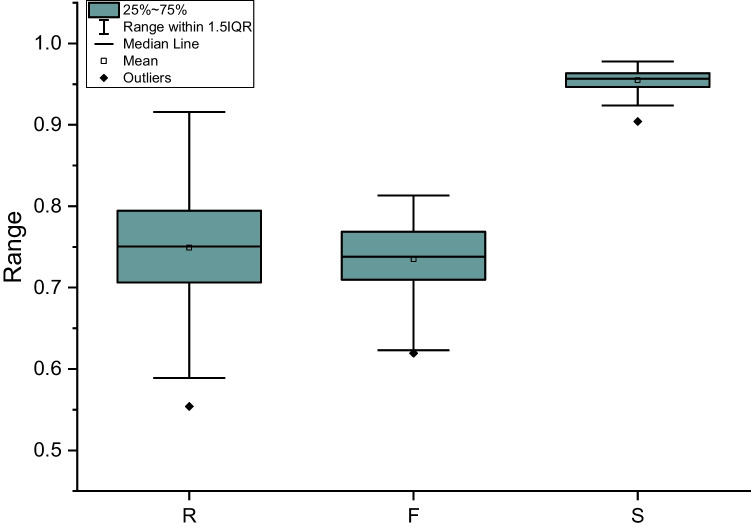
Boxplot representation of morphometric parameters roundness, form factor, and sphericity as retrieved with optical method. *R* and *F* results are very similar while *S* deviates substantially

Each clast was dropped 20 times and photographed during free fall. The longest axis in any of the 20 photos per sample was used for *V*_o_ calculation, while the short axis represents the average value from 20 values for each clast. For each sample, *R* (roundness), *F* (form factor), and *S* (sphericity) parameters were calculated (Fig. 3; see Supplementary Material for full dataset) and show that the distribution’s standard deviation of values is highest for *R* (0.06) and smallest for *S* (0.01). This is also observable for the average standard deviation (for each clast over 20 measurements, see Supplementary Material) for *R*, implying that this parameter is very sensitive to clast shape anisotropies that become apparent over 20 photographs.

### Pycnometry, density, and porosity

**Fig. 4:**
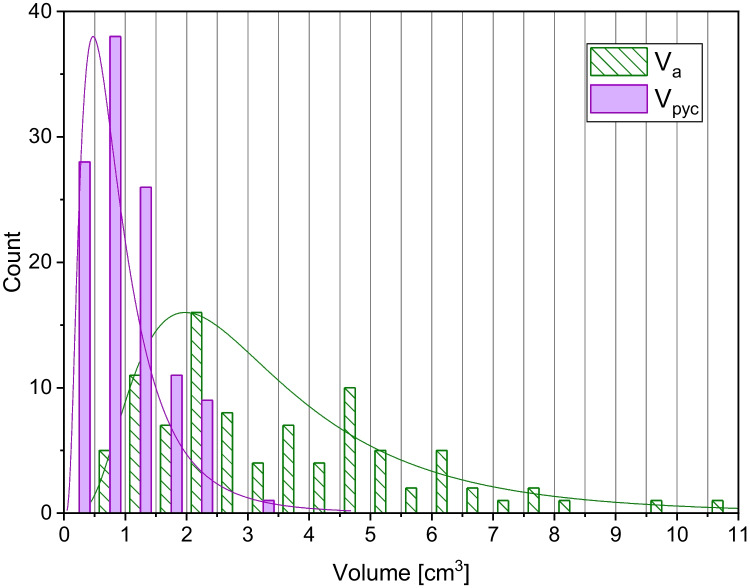
Frequency distributions of Archimedean (*V*_a_) and pycnometry (*V*_pyc_) volume showing the expected apparently reduced *V*_pyc_ due to the porous nature of the Santorini clasts. Bin size 0.5 cm^3^

**Fig. 5:**
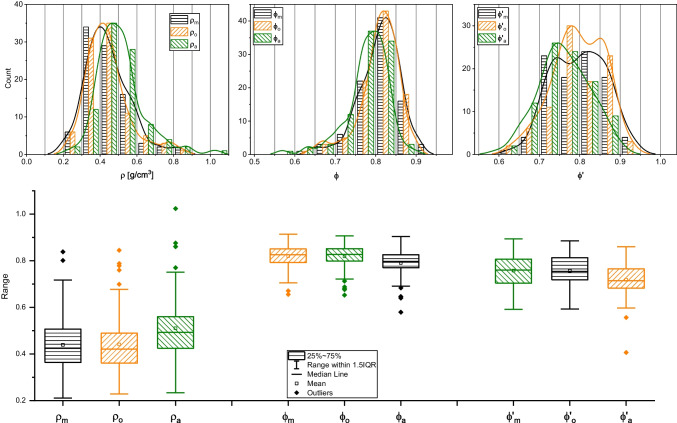
Boxplots and frequency distributions of bulk density (a, bin size 0.1), bulk porosity (b, bin size 0.05), and open porosity (c, bin size 0.05) values as calculated from the three-volume datasets (*V*_m_, *V*_o_, and *V*_a_ in Fig. 1)

Pycnometry was used on individual clasts (*n* = 794) as well as sample powder pulverized as indicated above to determine two kinds of values, the pycnometric volume *V*_pyc_ (conceptually the volume of solid phase + isolated clasts) and the dense rock equivalent (DRE or *ρ*_DRE_). While *V*_a_ values of our sample set were as high as 11 cm^3^, *V*_pyc_ values were significantly smaller, never higher than 3.5 cm^3^ and with mean and median values lower than 1 cm^3^ (Fig. 4). This large difference between *V*_a_ and *V*_pyc_ is reasonable for rhyodacitic pumices, in which connected pores represent the vast majority (more than 80%) of total pores (Colombier et al. 2017b). *V*_pyc_ together with *V*_m_, *V*_o_, or *V*_a_ values, on the other hand, were used to calculate connected porosity values (*ϕ*′_m_, *ϕ*′_o_, *ϕ*′_a_) with Eq. 7 and are reported in Fig. 5a. The *ρ*_DRE_ of powdered pumices reveal a value of 2.43 g/cm^3^, which was used to calculate bulk density values (*ρ*_m_, *ρ*_o_, *ρ*_a_) from Eq. 8 and bulk porosity values (*ϕ*_m_, *ϕ*_o_, *ϕ*_a_) from Eq. 9. These values are reported in Fig. 5 b and c.

We observed significant textural heterogeneity with open porosity values (*ϕ*′) between 20 and 80 vol.% and bulk porosity values as high as 92 vol.%. Figure 5 clearly conveys how different volume determination techniques lead to similar but not identical *ϕ*′, *ϕ*, and *ρ* distributions.

### Humidity experiment for field validation

**Fig. 6:**
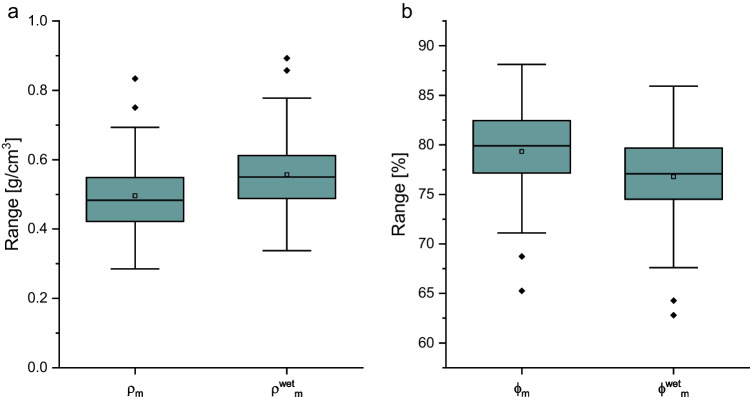
Boxplots of bulk density (**a**) and bulk porosity (**b**) values to show the limited impact of sample humidity. In both graphs, the left boxplot shows the variation for dry clasts and the right one for the same clasts after controlled wetting, respectively. All values have been calculated from volume *V*_m_ obtained with manual methodology

The influence of humidity on the volume assessment was investigated by comparing the weight of dry clasts and the same respective clast after controlled and repeatable wetting. Dry (*ϕ*_m_) and humid (${\phi}_{\textrm{m}}^{\textrm{wet}}$) porosities were calculated using *V*_m_, *ρ*_DRE_, and the dry and humid weight, respectively, and are reported in Fig. 6. While the average weight increase on wetting was up to 12.3% (0.3 g on average), the effective change of calculated porosity was 2.5% on average. These changes are not correlated to clast size and confirm the textural heterogeneity of the investigated Minoan clasts.

## Discussion

The textural characteristics of clasts from tephra deposits contain information about the state of magma at fragmentation, eruption intensity, and dispersal conditions. While deciphering the fine-scale information contained in pyroclastic deposits is challenging and is largely carried out in the laboratory (Douillet et al. 2018, 2019), we propose that additional measurements in the field allow for a rapid yet comparatively easy description of clast textures that are important for constraining magma textural heterogeneity, ultimately one of the principal parameters affecting eruption style.

We have compared different volume determination techniques of pumice lapilli from Santorini (Greece) in a laboratory study to reveal the meaningfulness and reliability of volume quantification via different approaches. To this end, clasts from the same sample sets have been subject to repetitive measurements. We have demonstrated that the porosity of pumice clasts can be reliably measured in the field at satisfying precision, and we address the following possible sources of inaccuracies:

1. Subjective sampling: The sample set was sampled by two individuals and their relative subsets reflect each other, therefore no subjective bias was revealed.
2. Manual length measurement with calipers: Three orthogonal axes were measured, and the related results were communicated to a second person. This way, ambiguities of sample orientation, especially for measuring the short and middle axes can be excluded. Electronic calipers avoid misreading of the effective length values. If mechanical calipers were used, the expected maximum misreading is 0.5 mm and the impact on the total volume calculation is small (<< 1 mm^3^).
3. Optical assessment of clast long and short axes: Initial experiments revealed that 20 repetitive measurements of one clast in free fall will guarantee the evaluation of the longest and shortest axes and a subsequent quantification of volume assuming a spheroidal shape which is very close to the one achieved through the manual method.
4. Archimedean volume assessment: Each clast was wrapped in parafilm (0.1–0.4 g, 0.17 g on average). Given its thin nature, the added volume contributed by the parafilm will affect the displaced amount of water and accordingly the clast volume by increasing it by 6.6% on average.
5. Clast humidity has been assessed for 50 randomly selected clasts. Dry and humid weight (after reproducible “water treatment”) has been assessed with a high-precision balance (10^−4^ g).

**Fig. 7:**
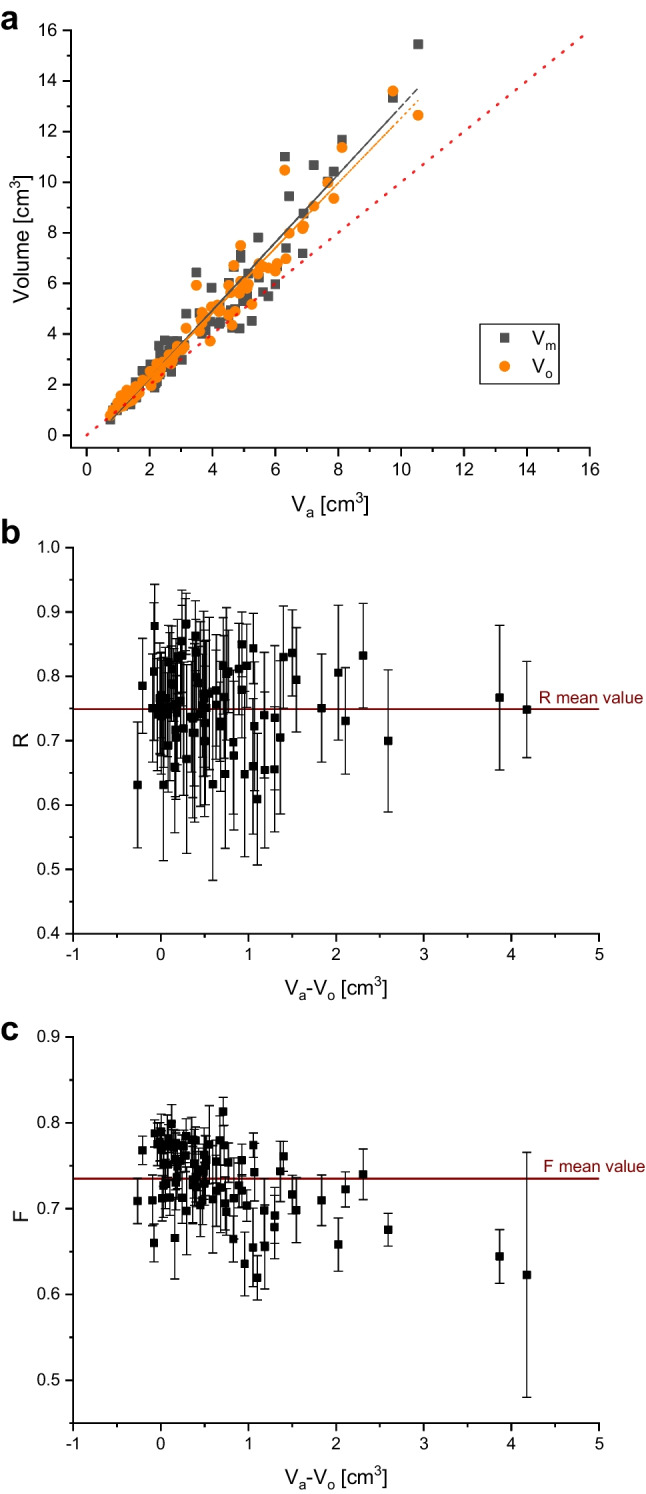
Relationship between manual (*V*_m_) and optical (*V*_o_) volumes to their respective Archimedean (*V*_a_) volume for Santorini pumices, showing the similarity between *V*_m_ and *V*_o_ with consistently higher values than *V*_a_ (**a**). The dashed line indicates the 1:1 ratio. Shape parameters *R* (**b**) and *F* (**c**) have been plotted to investigate possible clast shape effects on Δ*V*, the difference between optical and Archimedean volume values. While *R* shows no apparent effect of clast size and/or Δ*V* (**b**), *F* seems to show some negative correlation (**c**)

Concerning the evaluated volumes, all three approaches have limitations that must be considered. The parafilm wrapping will increase clast volume (*V*_a_) as material is added but the effect is reasonably lower (supposedly 6.6% on average) than any error introduced by assuming a regular geometrical clast shape, since *V*_m_ values are averagely 13% larger than *V*_a_ values (Figs. 1 and 4; Table 2). In Fig. 7a, it is possible to see that *V*_m_ and *V*_o_ show extremely similar results in both distribution and linearity, whereas values for *V*_a_ deviate linearly (as said, ca. 13% on average) from a 1:1 trend, positively correlated with clast size (Fig. 7a).

Since *V*_m_ and *V*_o_ rely on the approximation of clast shape to an ellipsoid or spheroid, respectively, we investigated shape anisotropy by determining the shape parameters *R*, *F*, and *S*. Figure 7b shows the variability of *F* and *R* when compared to the difference between *V*_a_ and *V*_o_ (Δ*V*) values. *R* values do not show correlation with size (or volume difference; Fig. 7b). It became apparent again that *R* is of limited meaningfulness as the standard deviation per clast (error bars) is as large as the variability of the average points. *F* values on the other hand show a more defined result cluster for most of the data set and a slightly negative correlation of Δ*V* and *F*. We have seen how three different techniques for volume determination produce consistent results with tolerable deviations of properties like density and porosity (Fig. 5).

**Fig. 8:**
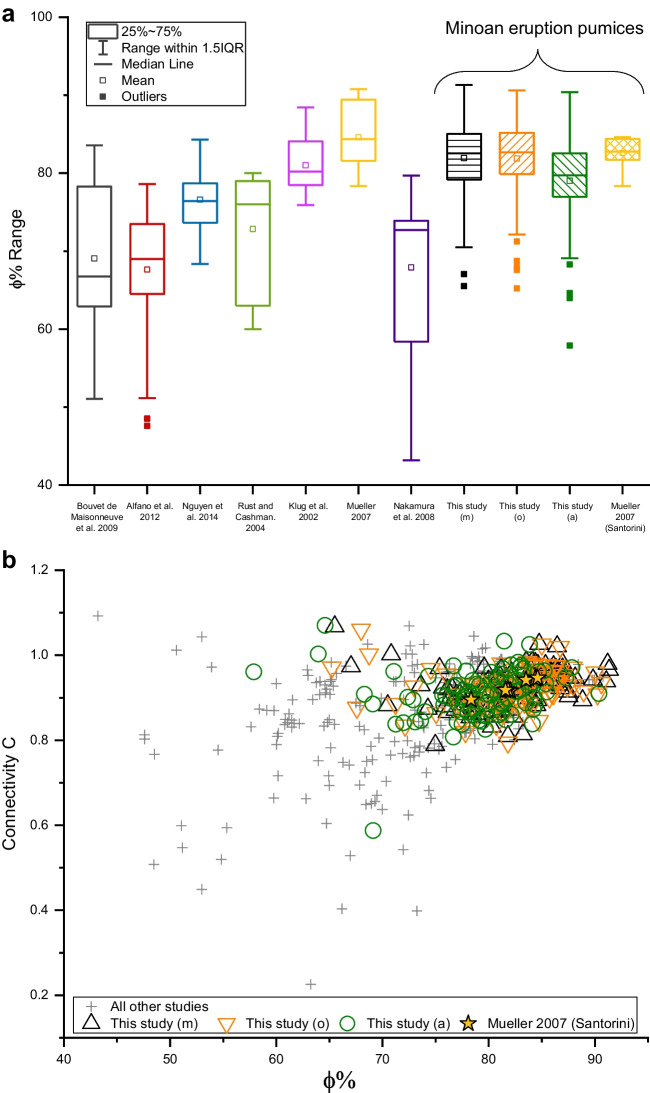
Range of total porosity values for published rhyodacitic pumice (the seven boxplots on the left) as compared to Santorini results from this study (see Fig. 4c) and Müller (2007) (**a**). The porosity-connectivity relationship (**b**) shows the apparent textural link between chemically controlled vesiculation and bubble growth on the one hand and the development of a permeable network which will ultimately affect eruption dynamics

The primary aim of this study was to test the robustness of field-based pyroclast volume determination, in order to allow for a holistic characterization of the deposits of volcanic explosions. To put our findings into context, we used data from a recent review (Colombier et al. 2017b) on rhyodacitic pumices. Figure 8a compares our porosity values (right side of the graph) to other tephra clasts from chemically comparable eruptions. It is evident that the differences between *ϕ*_m_, *ϕ*_o_, and *ϕ*_a_—as revealed in this study for Santorini—are smaller than the observed heterogeneity of rhyodacitic pumices in general. Moreover, to include open porosity values in our comparison, we can calculate connectivity *C* after as follows:

10 $$C=\frac{\varphi^{\prime }}{\varphi }$$

In Fig. 8b, we can observe the *C*-*ϕ* relationship of our dataset, compared with other datasets regarding rhyodacitic pumices. Here, the clusters of data concerning Archimedean, manual, and optical methodologies overlap each other, and their discrepancies look less marked if observed in the context of possible variability of porosity and connectivity values for this kind of pyroclasts, which is represented by all the datapoints in the graph. More interestingly, we can observe how the *C* and *ϕ* values of Santorini’s Minoan pumices calculated by Mueller et al. (2008) fall in the middle of our data cluster (star symbols in Fig. 8b). Additionally, the porosity distribution values determined by Mueller et al. 2008 (yellow boxplot on the right in Fig. 8a) are more similar to “our” *ϕ*_m_ and *ϕ*_o_ values than to our *ϕ*_a_ values. It is worth noting that Mueller et al. (2008) investigated cylindrical core samples of pumice extracted from larger blocks. Accordingly, in that study, the determination of volume (and consequently of porosity and density) was straightforward since the volume of a core sample was easily determined from its shape, but we observe that we can obtain comparable results without the need for core drilling.

### The manual method as a reliable field analysis tool

As the discrepancy between volumes obtained manually and with the Archimedean method is minor, the manual method has the potential to be used during field studies to quickly characterize pyroclasts. We suggest incorporating volume and porosity measurements in future field work, which would constrain the textural heterogeneity of the erupted pyroclasts and accordingly, eruption dynamics.

**Fig. 9:**
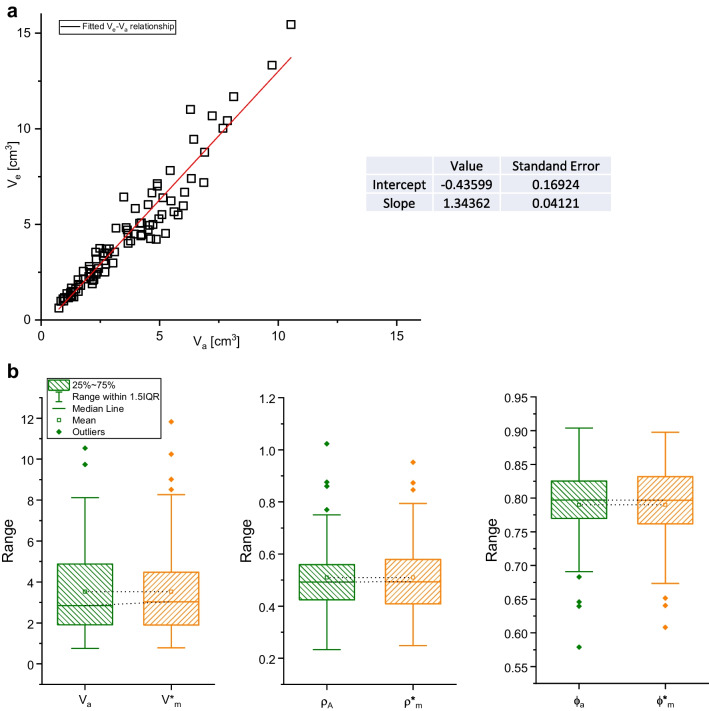
Linear fitting of manual and Archimedean volume values (**a**) and boxplots of volume, density, and porosity distributions as retrieved with Archimedean methodology or with the corrected manual methodology (**b**)

Since the discrepancy between manual and Archimedean methods was found to be of a linear nature and volume-dependent (see Fig. 7a), we are able to introduce a correction coefficient for field analyses. In Fig. 8a, we have plotted *V*_a_ and *V*_m_ data to interpolate them linearly, and from the values of such interpolation (reported in Fig. 9a as well), we can calculate the corrected volume ${V}_{\textrm{m}}^{\ast }$ values as follows:

11 $${V}_{\textrm{m}}^{\ast }=\frac{V_{\textrm{m}}-\textrm{Intercept}}{\textrm{Slope}}$$

From these values, we are able to calculate corrected values of density${\rho}_{\textrm{m}}^{\ast }$ and porosity${\varphi}_{\textrm{m}}^{\ast }$. In Fig. 9b, we can observe the distributions of corrected values versus Archimedean-derived values which look almost identical. In this way, we provide a possible correction protocol for fast and in-field characterization of rhyodacitic pumices. Such a correction factor would be reasonably valid in the case of eruptions that produced rhyodacitic pumices with characteristics, but a more universal validity needs to be confirmed by further studies using this method on pyroclasts having different compositional and textural characteristics, e.g., basaltic scoriae or pumices with andesitic/trachytic/dacitic compositions.

The possible influence of humidity on volume and porosity evaluation was found to be minor (Fig. 6). Although a moisture-driven weight increase of up to 12.3% was revealed, the resulting porosity values decreased by less than 3%. In the light of observed textural variability within samples from one eruption and assuming that all clasts will have absorbed similar percentages of water, the humidity effect on porosity evaluation is likely of negligible importance. However, local climate or season may variably affect clasts from different outcrops by providing more or less humidity, but it would be possible to tackle this problem by sampling a small number of samples to be weighed in the field and after drying, to assess the local influence of humidity.

## Conclusions

Magmatic explosive eruptions are driven by overpressure built up in pore space from volatile exsolution. The eruption dynamics depend on a combination of magma ascent conditions, resulting magma textures (porosity, permeability, etc.), and acting overpressure at fragmentation. In this study, we have tested three methodologies to assess the volume of lapilli-sized pyroclasts and evaluate their influence on the calculation of open porosity, bulk density, and bulk porosity. The measurement time required per clast ranges from approximately 15 s to 2 min. This laboratory-based comparative study has revealed that the differences in outcomes between the three methods are smaller than the natural variability of these parameters.

The manual method is a fast and reliable method that allows for investigation of juvenile pyroclasts in the field. As it can be performed directly in the field, without sample preparation and with just calipers and a balance, it is useful for rapid response description of deposits during crisis, as well as repeated investigations during prolonged eruptions. In addition, constraining the textural properties of a statistically meaningful set of pyroclasts directly in the field significantly reduces logistics and avoids any post-sampling clast shape alteration. Field-based porosity measurements represent a feasible and practical methodology that can be used to obtain an exhaustive description of volcanic deposits rapidly during field campaigns. Thus, we advocate for adding pyroclast porosity as a parameter to be measured directly in the field in future campaigns to constrain the range of magma heterogeneity in space and time.

### Supplementary Information

ESM 1 (XLSX 251 kb)

## Abbreviations

*V*_m_: Volume as determined with manual method (Eq. 1)
*V*_o_: Volume as determined with optical method (Eq. 2)
*V*_a_: Volume as determined with Archimedean method (Eq. 6)
*ϕ*^′ ^ (*ϕ*′_m_, *ϕ*′_o_ ,*ϕ*′_a_): Open porosity (also called connected porosity). Subscript letter indicates if such property was calculated using *V*_*m*_, *V*_*o*_, or *V*_*a*_ (Eq. 7)
*ρ * (*ρ*_m_, *ρ*_o_ ,*ρ*_a_): Bulk density. Subscript letter indicates if such property was calculated using *V*_*m*_, *V*_*o*_, or *V*_*a*_ (Eq. 8)
*ϕ * (*ϕ*_m_, *ϕ*_o_ ,*ϕ*_a_): Bulk porosity. Subscript letter indicates if such property was calculated using *V*_*m*_, *V*_*o*_, or *V*_*a*_ (Eq. 9)
*ρ*_DRE_: Density of the solid part of the clasts (dense rock equivalent) measured with pycnometry
*R*: Roundness calculated from photographs of clasts produced with optical method (Eq. 3)
*F*: Form factor calculated from photographs of clasts produced with optical method (Eq. 4)
*S*: Sphericity calculated from photographs of clasts produced with optical method (Eq. 5)
*C * (*C*_m_, *C*_o_, *C*_a_): Connectivity defined as the ratio of open porosity over the bulk porosity. Subscript letter indicates if such property was calculated from porosity values retrieved using manual, optical, or Archimedean method (Eq. 10)
${V}_{\textrm{m}}^{\ast },{\rho}_{\textrm{m}}^{\ast }$, ${\phi}_{\textrm{m}}^{\ast }$: Values of volume, bulk density, and porosity retrieved from volume correction as in Eq. 11
${\rho}_{\textrm{m}}^{\textrm{wet}},{\phi}_{\textrm{m}}^{\textrm{wet}}$: Bulk density and porosity as determined for wet clasts
